# Self‐reported hearing loss is associated with faster cognitive and functional decline but not diagnostic conversion in the ADNI cohort

**DOI:** 10.1002/alz.14252

**Published:** 2024-09-26

**Authors:** Alyssa A. Miller, Emily S. Sharp, Selena Wang, Yize Zhao, Adam P. Mecca, Christopher H. van Dyck, Ryan S. O'Dell, for the Alzheimer's Disease Neuroimaging Initiative (ADNI)

**Affiliations:** ^1^ Alzheimer's Disease Research Unit Yale University School of Medicine New Haven Connecticut USA; ^2^ Department of Psychiatry Yale University School of Medicine New Haven Connecticut USA; ^3^ Department of Neurology Yale University School of Medicine New Haven Connecticut USA; ^4^ Department of Biostatistics Yale University School of Public Health New Haven Connecticut USA; ^5^ Department of Biostatistics and Health Data Science Indiana University School of Medicine Indianapolis Indiana USA; ^6^ Department of Neuroscience Yale University School of Medicine New Haven Connecticut USA

**Keywords:** cognition, conversion, hearing loss, memory, neuropsychological testing

## Abstract

**INTRODUCTION:**

Hearing loss is identified as one of the largest modifiable risk factors for cognitive impairment and dementia. Studies evaluating this relationship have yielded mixed results.

**METHODS:**

We investigated the longitudinal relationship between self‐reported hearing loss and cognitive/functional performance in 695 cognitively normal (CN) and 941 participants with mild cognitive impairment (MCI) enrolled in the Alzheimer's Disease Neuroimaging Initiative.

**RESULTS:**

Within CN participants with hearing loss, there was a significantly greater rate of cognitive decline per modified preclinical Alzheimer's cognitive composite. Within both CN and MCI participants with hearing loss, there was a significantly greater rate of functional decline per the functional activities questionnaire (FAQ). In CN and MCI participants, hearing loss did not significantly contribute to the risk of progression to a more impaired diagnosis.

**DISCUSSION:**

These results confirm previous studies demonstrating a significant longitudinal association between self‐reported hearing loss and cognition/function but do not demonstrate an increased risk of conversion to a more impaired diagnosis.

**CLINICAL TRIAL REGISTRATION INFORMATION:**

NCT00106899 (ADNI: Alzheimer's Disease Neuroimaging Initiative, clinicaltrials.gov), NCT01078636 (ADNI‐GO: Alzheimer's Disease Neuroimaging Initiative Grand Opportunity, clinicaltrials.gov), NCT01231971 (ADNI2: Alzheimer's Disease Neuroimaging Initiative 2, clinicaltrials.gov), NCT02854033 (ADNI3: Alzheimer's Disease Neuroimaging Initiative 3, clinicaltrials.gov).

**Highlights:**

- Hearing loss is a potential modifiable risk factor for dementia.
- We assessed the effect of self‐reported hearing loss on cognition and function in the ADNI cohort.
- Hearing loss contributes to significantly faster cognitive and functional decline.
- Hearing loss was not associated with conversion to a more impaired diagnosis.

## 1 BACKGROUND

In the United States and around the world, the population continues to age, with the percentage of individuals over the age of 60 worldwide expected to double from 12% to 22% by 2050.^1^ As such, the incidence of age‐related disorders, such as dementia, is also expected to rise. Alzheimer's disease (AD) is the leading cause of dementia, with age being the most impactful risk factor.^2^ About 55 million individuals worldwide have some form of dementia, with that number expected to increase to 152 million by 2050.^2^, ^3^ In the United States, an estimated 6.7 million individuals are currently living with AD, a number expected to increase to 13.8 million by 2060.^2^ As such, it is critical to identify modifiable contributors to disease risk that may lead to interventions aimed at delaying or preventing the expression of AD.

Hearing loss has been identified as a potential risk factor for dementia.^4^, ^5^, ^6^, ^7^ Globally, about one‐third of individuals above the age of 65 are living with hearing loss, and 585 million individuals over the age of 65 are expected to develop hearing loss by 2050.^8^ This large proportion of the population may therefore be at greater risk for developing dementia,^3^ and studies have suggested that intervention, primarily the use of hearing aids, may mitigate this risk.^9^, ^10^ However, the observed mitigation of cognitive decline with hearing aid use has been largely based on analyses of datasets with self‐reported hearing loss and hearing aid use.^9^, ^10^ Such studies are confounded by differential rates of hearing aid use based on demographic and socioeconomic variables^11^ and a lack of information surrounding the frequency of use and efficacy of the intervention. To this point, although a recent multicenter randomized controlled trial showed no significant effect of hearing intervention in the primary analyses of the total cohort, a prespecified sensitivity analysis in the Atherosclerosis Risk in Communities (ARIC) cohort demonstrated hearing intervention may reduce 3‐year cognitive decline in older adults at increased risk for future decline.^12^ As such, investigations of hearing loss and risk for cognitive and functional decline are critical areas for study and intervention.

The existing literature presents a variable and complex relationship between hearing loss and cognition. Recent studies have demonstrated that hearing loss is associated with poorer cognitive test scores at baseline and across longitudinal evaluation and has identified hearing loss as a risk factor for accelerated cognitive decline and earlier onset of dementia.^4^, ^6^, ^13^, ^14^ In contrast, another study found that the association between hearing loss, dementia risk, and scores on longitudinal cognitive measures was reduced to non‐significance after adjusting for the interaction between age and follow‐up time of evaluation.^5^ Further, an increased risk of conversion to dementia from normal cognition in participants with treated hearing loss was observed in the National Alzheimer's Coordinating Center (NACC) Uniform Data Set.^15^ However, sensitivity analyses demonstrated no increased risk of conversion to dementia in a combined group of participants with treated and untreated hearing loss (compared to those without hearing loss). This study and others raise the question of whether hearing loss is a direct risk factor for conversion to dementia or whether hearing loss is associated with an increased risk only when combined with other variables.

Despite mixed findings, the literature overall suggests that hearing loss is associated with a worse performance on baseline and longitudinal cognitive testing scores, including measures of visual memory, which are less likely to be impacted by hearing ability.^16^ Yet, when studies have examined hearing loss as a risk for conversion to dementia the findings have been inconsistent. Some studies have suggested an increased risk for dementia, but only when demographic variables were not controlled for, and others have found no relationship.^5^, ^15^ It is therefore essential to better understand whether hearing loss is a potentially modifiable risk factor for cognitive impairment.

In this study, we investigated the relationship between self‐reported hearing loss and cognition using data collected from the Alzheimer's Disease Neuroimaging Initiative (ADNI). Our goals were 1. to assess the association between hearing loss and cognitive/functional performance at baseline and longitudinally, and 2. to investigate whether hearing loss increased the risk of progression to a more impaired diagnostic group.

## 2 METHODS

### 2.1 Study participants and design

Data were obtained from the ADNI database (adni.loni.usc.edu) on September 5, 2022. ADNI was launched in 2003 as a public–private partnership, led by Michael W. Weiner, MD. The primary goal of ADNI has been to test whether serial magnetic resonance imaging (MRI), positron emission tomography (PET), or other biological markers and clinical and neuropsychological assessment can be combined to measure the progression of mild cognitive impairment (MCI) and early AD. For up‐to‐date information, see www.adni‐info.org.

All participants gave their informed consent, and the study protocol was approved by the committee on human research at each participating institution. ADNI is a longitudinal observational study of aging that enrolls participants diagnosed as cognitively normal (CN), subjective memory concerns (SMC), MCI (both early and late stages), and AD dementia. Detailed information describing diagnostic criteria can be found at www.adni‐info.org. CN participants had no subjective memory concerns and an absence of objective impairment in cognition or function (Logical Memory II Story A score of ≥9 for 16 or more years of education, ≥5 for 8 to 15 years of education, or ≥3 for 0 to 7 years of education, Mini‐Mental State Examination [MMSE] score of 24 to 30, and Clinical Dementia Rating [CDR] scale score of 0). SMC participants self‐reported significant memory concerns but had an absence of objective impairment in cognition or function. Early MCI (EMCI) subjects had a subjective memory concern, mildly abnormal memory performance (Logical Memory II Story A score of 9 to 11 for 16 or more years of education, 5 to 9 for 8 to 15 years of education, or 3 to 6 for 0 to 7 years of education, MMSE score of 24 to 30, and a CDR scale score of 0.5), and preserved functional performance such that a diagnosis of AD dementia could not be made. Late MCI (LMCI) subjects had a subjective memory concern, memory performance that was abnormal and below that of EMCI subjects (Logical Memory II Story A score of ≤8 for 16 or more years of education, ≤4 for 8 to 15 years of education, or ≤2 for 0 to 7 years of education, MMSE score of 24 to 30, and CDR scale score of 0.5), and preserved functional performance such that a diagnosis of AD dementia could not be made.^17^ AD dementia subjects had a subjective memory concern, abnormal memory performance (Logical Memory II Story A score of ≤ 8 for 16 or more years of education, ≤ 4 for 8 to 15 years of education, or ≤2 for 0 to 7 years of education, MMSE score of 20 to 26, CDR scale score of 0.5 or 1.0), and functional impairment meeting National Institute of Neurological and Communicative Disorders and Stroke/Alzheimer's Disease and Related Disorders Association (NINCDS/ADRDA) criteria for probable AD.^18^ For all analyses, EMCI and LMCI participants were combined into a single MCI group, and participants with SMC were included in the CN group. Diagnostic categorization at each visit was determined by the aforementioned criteria, and a determination of diagnostic conversion was left to the discretion of the primary investigator (PI) at each ADNI site, based on the foregoing criteria and clinical judgment. Data collected during the ADNI 1, Grand Opportunity (GO), 2, and 3 cohorts were used for all analyses.

**RESEARCH IN CONTEXT**

1. **Systematic review**: Hearing loss has been identified as a major potentially modifiable risk factor for dementia. Studies evaluating this relationship, however, have yielded mixed results.
2. **Interpretation**: In the ADNI sample of convenience, we demonstrated that self‐reported hearing loss was associated with faster cognitive and functional decline in 695 participants with normal cognition and 941 participants with mild cognitive impairment but was not associated with an increased risk of conversion to a more impaired diagnostic stage.
3. **Future directions**: Further study is needed to better understand the risk hearing loss confers on conversion to a more impaired diagnostic stage and how treatment with hearing aids might mitigate this risk. Given the pathologic heterogeneity of the ADNI cohort (despite a likely selection bias toward Alzheimer's disease), it would be of great interest for future studies to investigate the effect of hearing loss on different etiologies and stages (including preclinical Alzheimer's disease) of cognitive impairment.

### 2.2 Outcomes

The presence of hearing loss was determined from participants’ medical histories, which were verbally reported by caregivers and/or self‐reported by the participants at the first screening visit. Individuals with self‐reported hearing loss or mention of hearing aid use to their study coordinator during collection of their medical history were classified as having clinically significant hearing loss, and all others were classified as having no hearing loss. The presence and severity of hearing loss were not measured by objective testing. Mitigation of hearing loss with the use of hearing aids (as well as the frequency and effectiveness of use) was inconsistently reported and therefore not included as an outcome in the analyses. Participants who reported hearing loss after the initial screening visit were excluded to minimize reverse causation. Participants with documented hearing loss prior to baseline enrollment and more than one visit at which cognitive testing was completed were included in analyses. For all analyses, only participants with diagnoses of CN or MCI at baseline were included.

Linear mixed models were used to examine the association between self‐reported hearing loss and outcome variables. The primary cognitive outcomes included a modified version of the Preclinical Alzheimer's Cognitive Composite (mPACC) for participants with normal cognition at baseline and the Alzheimer's Disease Assessment Scale‐cognitive subscale (ADAS‐Cog‐11) in participants with MCI at baseline. The primary functional outcome for both CN and MCI participants was the Functional Activities Questionnaire (FAQ).^19^ The mPACC represents the sum of four standardized *z*‐scores of the Alzheimer Disease Assessment Scale–Cognitive Subscale Delayed Word Recall, Logical Memory Delayed Recall (LM Delayed Recall), MMSE, and (log‐transformed) Trail Making Test Part B Time to Completion (Trails B),^20^, ^21^ with lower scores indicating greater impairment. The ADAS‐Cog‐11 is a composite cognitive battery consisting of 11 tasks (both participant‐completed testing and observer‐based measurements) assessing memory, language, and praxis. It is scored on a scale of 0 to 70, with higher scores indicating greater impairment.^22^ Exploratory cognitive and functional outcomes in participants with both CN and MCI diagnoses at baseline included the CDR scale sum of boxes (CDR‐sb),^23^, ^24^ the MMSE,^25^ the Rey Auditory Verbal Learning Test (RAVLT) immediate score,^26^, ^27^ RAVLT learning score,^26^, ^27^ RAVLT percent forgetting,^26^, ^27^ LM Delayed Recall,^28^ digit symbol substitution (DSS) test score,^29^ Trails B score,^30^ a composite score of memory (ADNI‐Mem),^31^ and a composite score of executive function (ADNI‐EF).^32^ RAVLT immediate scores were calculated as a sum of Trials 1 to 5, RAVLT learning scores were calculated as Trial 5 − Trial 1, and RAVLT percent forgetting was calculated as (Trial 5 − Delayed)/(Trial 5). For survival analyses, an additional primary outcome of visit diagnosis was used to determine whether a participant converted from CN to MCI or AD dementia, or from MCI to AD dementia. Age, sex, years of education, apolipoprotein E (*APOE*) ɛ4 copy number, and baseline ADAS‐Cog‐11 scores were utilized to characterize the study cohort (Table 1) and included as covariates in all statistical models.

**TABLE 1:** Participant demographics and baseline neuropsychological testing.

	CN				MCI			
	Hearing loss	No hearing loss	Effect size	*p*	Hearing loss	No hearing loss	Effect size	*p*
Participants (%)	134 (19.3)	561 (80.7)	–	–	211 (22.4)	730 (77.6)	–	–
Age	75.2 ± 6.2	72.5 ± 6.0	0.45	<0.001^*^	76.2 ± 6.5	72.1 ± 7.5	0.55	<0.001^*^
Sex (% male)	84 (62.7)	235 (41.9)	0.17	<0.001^*^	162 (76.8)	398 (54.4)	0.19	<0.001^*^
Education	17.0 ± 2.3	16.4 ± 2.6	0.26	0.007^*^	16.4 ± 2.7	15.9 ± 2.8	0.17	0.026^*^
*APOE* genotype (%)			0.02	0.88			0.04	0.44
ɛ3ɛ3	91 (68.4)	385 (69.4)	–	–	109 (53.4)	344 (48.5)	–	–
ɛ3ɛ4	39 (29.3)	154 (27.7)	–	–	73 (35.8)	286 (40.3)	–	–
ɛ4ɛ4	3 (2.3)	16 (2.9)	–	–	22 (10.8)	79 (11.1)	–	–
Race (%)			0.09	0.19			0.06	0.57
White	128 (95.5)	501 (89.3)	–	–	202 (96.2)	681 (93.5)	–	–
Black	4 (3.0)	36 (6.4)	–	–	6 (2.9)	22 (3.0)	–	–
Asian	2 (1.5)	10 (1.8)	–	–	1 (0.5)	15 (2.1)	–	–
Other or multiple	0 (0)	14 (2.5)	–	–	1 (0.5)	10 (1.4)	–	–
Hispanic ethnicity (%)	4 (3.0)	22 (3.9)	0.02	0.61	4 (1.9)	28 (3.9)	0.05	0.17
mPACC	−0.21 ± 2.63	0.03 ± 2.58	0.09	0.33	−5.86 ± 3.58	−5.75 ± 3.92	0.03	0.70
ADAS‐Cog‐11	6.2 ± 2.9	5.6 ± 2.9	0.20	0.04^*^	10.1 ± 4.2	10.2 ± 4.6	0.03	0.75
CDR‐sb	0.03 ± 0.13	0.04 ± 0.14	0.07	0.47	1.6 ± 1.0	1.5 ± 0.9	0.15	0.08
MMSE	29.03 ± 1.2	29.11 ± 1.1	0.07	0.46	27.5 ± 1.8	27.6 ± 1.8	0.06	0.44
RAVLT Immediate	44.7 ± 9.7	45.6 ± 9.8	0.10	0.32	32.7 ± 9.7	34.8 ± 10.8	0.20	0.008^*^
RAVLT Learning	6.1 ± 2.4	6.1 ± 2.3	0.01	0.96	3.9 ± 2.4	4.1 ± 2.6	0.08	0.31
RAVLT Percent Forgetting	36.1 ± 28.7	34.8 ± 27.3	0.05	0.63	60.7 ± 30.3	59.9 ± 35.5	0.02	0.77
Logical Memory Delayed Recall	13.5 ± 3.3	13.0 ± 3.4	0.16	0.10	6.1 ± 3.4	5.7 ± 3.5	0.11	0.16
Trails B	84.2 ± 43.0	81.8 ± 42.7	0.06	0.57	116.1 ± 63.6	115.8 ± 66.2	0.004	0.96
Digit Symbol Substitution Test	44.4 ± 8.4	45.8 ± 10.7	0.14	0.40	36.3 ± 10.0	37.0 ± 11.6	0.07	0.55
ADNI‐Mem	0.98 ± 0.56	1.03 ± 0.55	0.08	0.046^*^	0.11 ± 0.60	0.18 ± 0.69	0.11	0.13
ADNI‐EF	0.82 ± 0.83	0.88 ± 0.83	0.07	0.50	0.19 ± 0.85	0.21 ± 0.90	0.03	0.74
FAQ	0.42 ± 1.51	0.15 ± 0.59	0.31	0.047^*^	3.40 ± 3.92	3.23 ± 4.23	0.04	0.61

*Note*: Data for continuous variables are mean ± SD. Data for participants, sex, race, ethnicity, and *APOE* copy number are *n* (percentage). Unpaired *t* tests (for continuous variables) and *χ^2^* tests (for categorical variables) were used to compare baseline demographic and cognitive measures between CN and MCI participants with and without hearing loss. Effect sizes are denoted by Cohen's *d* for continuous variables, phi coefficient for categorical variables with two groups (sex, ethnicity), or Cramer's V for categorical variables with more than two groups (*APOE* genotype, race).

Abbreviations: ADAS‐Cog‐11, Alzheimer's Disease Assessment Scale‐Cognitive Subscale; ADNI‐EF, composite executive function score; ADNI‐Mem, composite memory score; *APOE*, apolipoprotein E; CDR‐sb, Clinical Dementia Rating scale–sum of boxes; CN, cognitively normal; FAQ, Functional Activities Questionnaire; MCI, mild cognitive impairment; MMSE, Mini‐Mental Status Examination; mPACC, modified Preclinical Alzheimer's Cognitive Composite; RAVLT, Rey Auditory Verbal Learning Test.

* Denotes significant group differences (*p* < 0.05) between participants with hearing loss and those without hearing loss within that diagnostic group (CN or MCI).

### 2.3 Statistical analyses

Independent analyses were performed for each baseline cognitive diagnosis: CN and MCI. A dichotomous variable was assigned as either positive for hearing loss prior to screening or negative for hearing loss prior to screening, per self‐report. A comparison of baseline characteristics between participants with and without hearing loss included age, sex, education, *APOE* ɛ4 copy number, and all cognitive outcome variables previously described. Simple *t* tests were used for continuous variables and *χ^2^* testing for categorical variables.

To determine whether differences in longitudinal cognitive decline were dependent on the presence of self‐reported hearing loss, separate repeated‐measures linear mixed models were used with different cognitive and functional measures as the outcome variable (described above) and the interaction of hearing loss and time (in months) from ADNI baseline visit as the main explanatory variable. Age, sex, education, *APOE* ɛ4 copy number, baseline ADAS‐Cog‐11, and a random intercept of participant ID (to control for repeated measures of the same participant) were included as covariates. Separate models were used for participants with baseline diagnoses of CN and MCI. *F* statistics, parameter estimates, and *p* values were reported for the main explanatory variable of the hearing loss×time interaction term in each model. The main effects of hearing loss, time (visit number in months), age, sex, education, *APOE* ɛ4 copy number, and baseline ADAS‐Cog‐11 are reported as exploratory measures in the Supplemental Material.

Survival curves of conversion from CN to MCI or AD dementia and conversion from MCI to AD dementia were calculated using the Kaplan–Meier method. Separate Cox proportional hazards models were used to assess the risk of conversion based on the presence or absence of self‐reported hearing loss, with the same covariates as described above. The confidence level for statistical inference was 95% (*p* < 0.05). No corrections for multiple comparisons were applied. Statistical analyses were performed using SPSS Statistics Version 28.0 (IBM Corp.).

## 3 RESULTS

### 3.1 Participant characteristics

Of the 695 participants who were clinically categorized as CN at baseline, 134 (19.3%) were categorized as having hearing loss, per self‐report. The majority of CN participants were white (90.5%) and non‐Hispanic (96.3%). Compared to participants with no hearing loss, CN participants with hearing loss were significantly older (75.2 ± 6.2 vs 72.5 ± 6.0 years, *t* (693) = −4.63, *p *< 0.001, Cohen's *d* = 0.45), were more likely to be male (62.7% vs 41.9%, *χ^2^* = 18.84, *p *< 0.001, phi coefficient = 0.17), and had more years of education (17.0 ± 2.3 vs 16.4 ± 2.6, *t* (693) = −2.69, *p *< 0.007, Cohen's *d* = 0.26; Table 1). CN participants with hearing loss also demonstrated significantly worse performance on baseline ADAS‐Cog‐11 (6.2 ± 2.9 vs 5.6 ± 2.9, *t* (692) = −2.06, *p *= 0.04, Cohen's *d* = 0.20), ADNI‐Mem (0.98 ± 0.56 vs 1.03 ± 0.55, *t* (693) = 0.88, *p *= 0.046, Cohen's *d* = 0.08), and FAQ (0.42 ± 1.51 vs 0.15 ± 0.59, *t* (693) = −3.27, *p *= 0.047, Cohen's *d* = 0.31) scores. CN participants without hearing loss were followed for an average of 59 months (range 6 to 186 months) with an average of five visits for cognitive assessment (range 2 to 15 visits) while those with hearing loss were followed for an average of 66 months (range 6–180 months) with an average of six visits for cognitive assessment (range 2 to 14 visits). The number of observations (*n*) for each primary outcome measured at each visit by baseline diagnosis and hearing loss status is reported in Table S1.

Of the 941 participants who had a diagnosis of MCI at baseline, 211 (22.4%) were categorized as having hearing loss, per self‐report. Similar to CN participants, the majority of participants with MCI were White (93.8%) and non‐Hispanic (96.6%). Compared to participants with no hearing loss, MCI participants with hearing loss were significantly older (76.2 ± 6.5 vs 72.1 ± 7.5, *t* (936) = −7.03, *p *< 0.001, Cohen's *d* = 0.55), were more likely to be male (76.8% vs. 54.4%, *χ^2^* = 33.65, *p *< 0.001, phi coefficient = 0.19), and had more years of education (16.4 ± 2.7 vs 15.9 ± 2.8, *t* (939) = −2.22, *p *< 0.026, Cohen's *d* = 0.17; Table 1). MCI participants with hearing loss also demonstrated significantly worse performance on baseline RAVLT immediate (32.7 ± 9.7 vs 34.8 ± 10.8, *t* (939) = 2.54, *p *= 0.008, Cohen's *d* = 0.20) and FAQ (3.40 ± 3.92 vs 3.23 ± 4.23, *t* (929) = −0.50, *p *= 0.02, Cohen's *d* = 0.04) scores. All other demographic variables and baseline cognitive testing scores were not significantly different between participants with and without hearing loss with either diagnoses of CN or MCI at baseline (Table 1). MCI participants without hearing loss were followed for an average of 49 months (range 6 to 196 months) with an average of 6 visits for cognitive assessment (range 2 to 17 visits) while those with hearing loss were followed for an average of 51 months (range 6 to 186 months) with an average of six visits for cognitive assessment (range 2 to 17 visits).

### 3.2 Association between self‐reported hearing loss and longitudinal cognitive performance: linear mixed models

**FIGURE 1:**
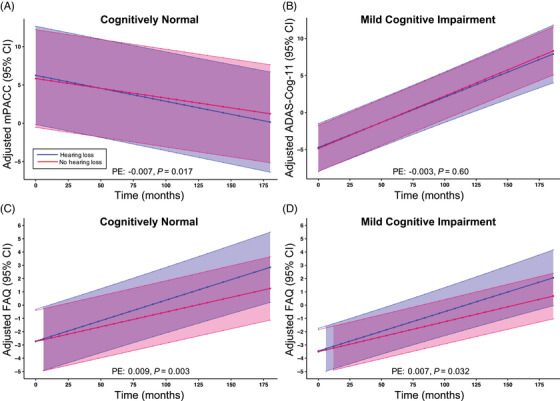
Change in cognition and function over time for participants with and without hearing loss. (A) Change in adjusted mPACC with 95% CI over time in participants with a baseline diagnosis of CN. (B) Change in adjusted ADAS‐Cog‐11 with 95% CI over time in participants with a baseline diagnosis of MCI. (C) Change in adjusted FAQ with 95% CI over time in participants with a baseline CN diagnosis. (D) Change in adjusted FAQ with 95% CI over time in participants with a baseline MCI diagnosis. Repeated‐measures linear mixed models with mPACC (A), ADAS‐Cog‐11 (B), or FAQ (C, D) as the outcome variable and the interaction of hearing loss and time as the main explanatory variable were used to determine the relationship between hearing loss, cognition, and time. Age, sex, education, *APOE* ɛ4 copy number, baseline ADAS‐Cog‐11, and a random intercept were included as covariates. Adjusted mPACC, ADAS‐Cog‐11, and FAQ scores represent an estimated mean when sex = male, *APOE* ɛ4 copy number = 2, baseline ADAS‐Cog‐11 = 0, age = 0, and education = 0. PEs and *p* values are reported for the main explanatory variable of the hearing loss×time interaction term from the original linear mixed models. ADAS‐Cog‐11, Alzheimer's Disease Assessment Scale‐Cognitive Subscore; CI, confidence interval; CN, cognitively normal; FAQ, Functional Activities Questionnaire; MCI, mild cognitive impairment; mPACC, modified Preclinical Alzheimer's Cognitive Composite; *APOE*, apolipoprotein E; PE, parameter estimate.

The association between self‐reported hearing loss and change in cognition and functionality over time was investigated using a linear repeated‐measures mixed‐effects model with presence of hearing loss (hearing loss vs no hearing loss) as the main explanatory variable and change in the mPACC score (for CN participants at baseline), change in the ADAS‐Cog‐11 score (for MCI participants at baseline), or change in the FAQ (for both CN and MCI participants at baseline) over time as the outcome variable (Table 2). Covariates included age, sex, education, *APOE* ɛ4 copy number, and baseline ADAS‐Cog‐11. Within the CN sample, there was a significantly greater rate of decline in cognition over time in participants with hearing loss (compared to those without hearing loss) as measured by the mPACC (*F* = 5.67, PE = −0.007, *p *= 0.017; Table 2, Figure 1A). Within the MCI sample, there was no significant interaction between hearing loss and time on the primary outcome of ADAS‐Cog‐11 (*F* = 0.27, PE = −0.003, *p *= 0.60; Table 2, Figure 1B). In both the CN and MCI samples, there was a significant association between hearing loss and time on the FAQ (CN: *F* = 8.93, PE = 0.009, *p *= 0.003; MCI: *F* = 4.60, PE = 0.007, *p* = 0.032; Table 2, Figure 1C,D). The main effects of hearing loss, time (visit number in months), age, sex, education, *APOE* ɛ4 copy number, and baseline ADAS‐Cog‐11 are reported as exploratory measures in Tables S2 to S4.

**TABLE 2:** Association between hearing loss and change in cognition and function over time.

	CN at baseline			MCI at baseline		
	*F*	Parameter estimate	*p*	*F*	Parameter estimate	*p*
mPACC	5.67	−0.007	0.017^*^	1.36	−0.004	0.224
ADAS‐Cog‐11	2.08	0.005	0.15	0.27	−0.003	0.60
FAQ	8.93	0.009	0.003^*^	4.60	0.007	0.032^*^

*Note*: To determine whether differences in cognitive and functional decline were associated with the presence of hearing loss, separate repeated‐measures liner mixed models were used with either mPACC, ADAS‐Cog‐11, or FAQ scores as the outcome variable and the interaction of hearing loss and time as the main explanatory variable. Age, sex, education, *APOE* ɛ4 copy number, baseline ADAS‐Cog‐11, and a random intercept were included as covariates. Separate models were used for participants with baseline diagnoses of CN and MCI. F statistics, parameter estimates, and *p* values are reported for the main explanatory variable of the hearing loss×time interaction term. Time is documented in months for these models.

Abbreviations: ADAS‐Cog‐11, Alzheimer's Disease Assessment Scale‐Cognitive Subscore; CN, cognitively normal; MCI, mild cognitive impairment; mPACC, modified Preclinical Alzheimer's Cognitive Composite; *APOE*, apolipoprotein E.

* *p* < 0.05.

Exploratory analyses on the association between hearing loss and other measures of cognition revealed that within the CN sample, there was a significant interaction between hearing loss and time on CDR‐sb (*F* = 7.30, PE = 0.003, *p *= 0.007), MMSE (*F* = 13.74, PE = −0.007, *p *< 0.001), RAVLT immediate (*F* = 6.18, PE = −0.02, *p *= 0.013), RAVLT percent forgetting (*F* = 5.60, PE = 0.08, *p *= 0.02), and ADNI‐Mem (*F* = 4.19, PE = −0.001, *p *= 0.04) scores (Table S5, Figure S1A–J). In all models with cognitive outcomes, the interaction between hearing loss and time was in the expected direction (worsening performance over time). Within the MCI sample, there was a significantly greater rate of decline in cognition over time in participants with hearing loss (compared to those without hearing loss) as measured by RAVLT percent forgetting (*F* = 7.61, PE = 0.13, *p *= 0.006), LM Delayed Recall (*F* = 39.32, PE = −0.02, *p *≤ 0.001), and ADNI‐Mem (*F* = 4.29, PE = −0.001, *p *= 0.04) scores (Table S5, Figure S2A–J). There was no demonstrated interaction between hearing loss and time on all other cognitive outcome measures in either the CN or MCI samples (Table S5).

### 3.3 Association between self‐reported hearing loss and diagnostic conversion: Cox hazards models

The association between self‐reported hearing loss and risk of progression to a more impaired diagnostic group was investigated using Cox proportional hazard models with hearing loss as the main explanatory variable. Age, sex, education, *APOE* ɛ4 copy number, and baseline ADAS‐Cog‐11 were included as covariates. Out of 695 participants with a baseline diagnosis of CN and at least one follow‐up visit, 126 (18.1%) progressed to a diagnosis of MCI or AD dementia (570 censored). Of note, 121 (17.4%) progressed from a diagnosis of CN to MCI, while 5 (0.7%) progressed from a diagnosis of CN to AD dementia. Out of 941 participants with a baseline diagnosis of MCI and at least one follow‐up visit, 351 (37.3%) progressed to a diagnosis of dementia (586 censored).

**FIGURE 2:**
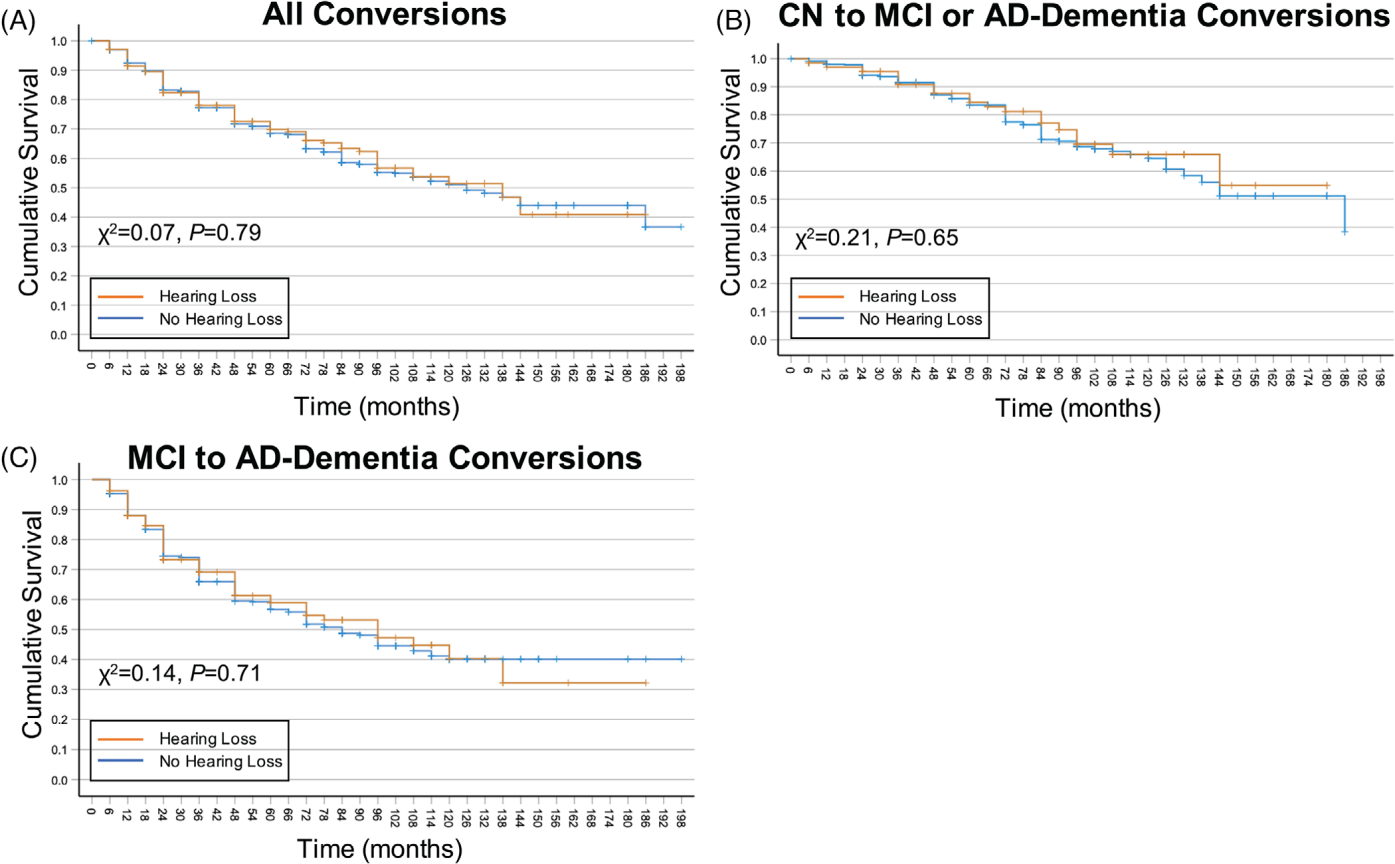
Risk of hearing loss on conversion to a more impaired diagnostic group. Survival curves were generated using the Kaplan–Meier method. Survival curves were generated for all conversion events (A; CN to MCI or AD dementia and MCI to AD dementia), CN to MCI or AD‐dementia conversion events only (B), and MCI to AD dementia conversion events only (C). Orange lines represent participants with hearing loss, and blue lines represent participants without hearing loss. *χ^2^* values and associated *p* values were derived using log‐rank (Mantel–Cox) test of equality of survival distributions. AD, Alzheimer's disease; ADAS‐Cog‐11, Alzheimer's Disease Assessment Scale‐Cognitive Subscale; CN, cognitively normal; MCI, mild cognitive impairment.

In a pooled sample of all participants, hearing loss did not significantly contribute to the risk of progression to a higher level of cognitive impairment (OR = 0.85, 95% CI = 0.68 to 1.07, *p *= 0.16; Table 3, Figure 2A). There was a significantly increased risk of progression to a more impaired diagnostic group conferred by age (OR = 1.04, 95% CI = 1.02 to 1.07, *p *< 0.001), baseline ADAS‐Cog‐11 (OR = 1.19, 95% CI = 1.17 to 1.21, *p *< 0.001), and *APOE* ɛ4 copy number (one copy: OR = 1.84, 95% CI = 1.51 to 2.25, *p * < 0.001; two copies: OR = 2.72, 95% CI 2.00 to 3.69, *p *< 0.001; Table 3).

**TABLE 3:** Risk of hearing loss and demographic variables on conversion to a more impaired diagnostic group.

	All conversions			CN to MCI or AD dementia			MCI to AD dementia		
	OR	95% CI	*p*	OR	95% CI	*p*	OR	95% CI	*p*
Hearing loss	0.85	0.68 to 1.07	0.16	0.69	0.44 to 1.09	0.11	0.94	0.72 to 1.22	0.62
Age	1.04	1.02 to 1.05	<0.001^*^	1.07	1.04 to 1.11	<0.001^*^	1.03	1.01 to 1.05	<0.001^*^
Female sex	0.85	0.70 to 1.03	0.09	0.98	0.67 to 1.43	0.91	0.81	0.64 to 1.01	0.06
Education	1.00	0.97 to 1.04	0.88	0.95	0.89 to 1.01	0.09	1.02	0.98 to 1.06	0.31
*APOE* ɛ4 (1 copy)	1.84	1.51 to 2.25	<0.001^*^	1.43	0.97 to 2.10	0.07	1.97	1.55 to 2.50	<0.001^*^
*APOE* ɛ4 (2 copies)	2.72	2.00 to 3.69	<0.001^*^	1.97	0.71 to 5.44	0.19	2.60	1.86 to 3.64	<0.001^*^
Baseline ADAS‐Cog‐11	1.19	1.17 to 1.21	<0.001^*^	1.16	1.10 to 1.23	<0.001^*^	1.17	1.15 to 1.20	<0.001^*^

*Note*: Three separate Cox proportional hazards models were used to assess the risk of conversion to a more impaired diagnostic group (from CN to MCI or AD‐dementia, MCI to AD‐dementia, or all conversion events) based on hearing loss and baseline demographic variables. Age, sex, education, *APOE* ɛ4 copy number, and baseline ADAS‐Cog‐11 were included as covariates. Odds ratios, 95% confidence intervals, and *p* values are reported for all variables in the model. References for categorical variables included no hearing loss, male sex, and 0 copies of *APOE* ε4.

Abbreviations: AD, Alzheimer's disease; ADAS‐Cog‐11, Alzheimer's Disease Assessment Scale‐Cognitive Subscale; *APOE*, apolipoprotein E; CN, cognitively normal; MCI, mild cognitive impairment; OR, odds ratio.

* *p* < 0.05.

When analyses were restricted to participants with a diagnosis of CN at baseline, hearing loss did not significantly contribute to the risk of progression to MCI (OR = 0.69, 95% CI = 0.44 to 1.09, *p *= 0.11; Table 3, Figure 2B). Similarly, in participants with a diagnosis of MCI at baseline, hearing loss did not significantly contribute to the risk of progression to AD dementia (OR = 0.94, 95% CI = 0.72 to 1.22, *p *= 0.62; Table 3, Figure 2B). Within the CN sample, there was a significantly increased risk of age (OR = 1.07, 95% CI = 1.04 to 1.11, *p *< 0.001) and baseline ADAS‐Cog‐11 (OR = 1.16, 95% CI = 1.10 to 1.23, *p *< 0.001) on conversion to MCI (Table 3). Within the MCI at baseline sample, there was a significantly increased risk of age (OR = 1.03, 95% CI = 1.01 to 1.05, *p *< 0.001), baseline ADAS‐Cog‐11 (OR = 1.17, 95% CI = 1.15 to 1.20, *p *< 0.001), and *APOE* ɛ4 copy number (one copy: OR = 1.97, 95% CI = 1.55 to 2.50, *p *< 0.001; two copies: OR = 2.60, 95% CI 1.86 to 3.64, *p *< 0.001) on conversion to AD dementia (Table 3).

## 4 DISCUSSION

We investigated the role of self‐reported hearing loss on cognitive and functional performance at baseline and over longitudinal measurements and whether hearing loss predicted conversion to a more impaired diagnostic group. Our findings suggest that both CN and MCI participants with hearing loss performed worse on cognitive and functional measures at baseline and longitudinally compared to those without hearing loss. However, hearing loss did not increase the risk of converting to a more impaired diagnostic group.

### 4.1 Association between self‐reported hearing loss and baseline cognitive and functional performance

At baseline, in both the CN and MCI samples, individuals with self‐reported hearing loss were more likely to be older, male, and have a higher level of education. These findings are consistent with current literature suggesting older individuals are more likely to develop hearing loss^33^ and that males have a higher incidence of hearing loss than females.^34^ The association of hearing loss with higher education that we found is inconsistent with the current literature^35^ and may be due to sampling bias in this cohort of convenience.^36^ As previously described,^37^ the majority of this sample was composed of non‐Hispanic White participants, which limits the external validity and generalizability of the findings.

Specific to our hypothesis, CN individuals with self‐reported hearing loss performed worse on the ADAS‐Cog‐11 and ADNI‐Mem, suggesting baseline global and memory‐specific differences. This finding demonstrates that in cognitively normal individuals, those with hearing loss are already performing worse at baseline on global measures of cognition compared to cognitively normal individuals without hearing loss. In the MCI sample, individuals with self‐reported hearing loss performed worse only on RAVLT immediate scores, suggesting few cognitive differences in these individuals. It is important to note that the difference in learning during the auditory word list could be attributable to differences in hearing acuity, such that individuals with hearing loss may have had greater difficulty clearly hearing the individual words. However, this pattern was not observed in cognitively normal participants with hearing loss. Lastly, we observed a significant interaction between hearing loss and time on RAVLT percent forgetting (in both CN and MCI participants). Therefore, regardless of the number of words initially encoded, those with hearing loss recall proportionally fewer words after a 30‐min delay. Thus, differences in encoding at baseline due to potential differences in hearing acuity do not explain the temporal relationship between hearing loss and verbal memory.

In terms of daily functioning, CN participants with self‐reported hearing loss were found to have comparatively lower functional independence at baseline as measured by the FAQ. This baseline difference in functional status, however, was not observed in MCI participants with self‐reported hearing loss. Overall, although individuals with hearing loss demonstrate limited cognitive differences at baseline, our findings suggest that cognitively normal individuals with hearing loss may already be demonstrating greater functional difficulty in real‐world settings. However, as it has been suggested that three to five points on this scale is indicative of a minimal clinically important difference,^38^ an absolute difference of 0.27 between those cognitively normal participants with and without hearing loss could be interpreted as statistically, but not clinically, significant.

### 4.2 Association between self‐reported hearing loss and longitudinal cognitive and functional performance

When examining longitudinal cognitive and functional data, we selected global measures evaluating multiple domains of cognition and independent functioning, including the mPACC for individuals staged as CN, the ADAS‐Cog‐11 for those with MCI, and the FAQ in both groups. In the CN group, as predicted, those with self‐reported hearing loss performed significantly worse on the mPACC over time, indicating that in individuals with normal cognition, hearing loss predicts worse performance on a global composite designed to capture subtle cognitive changes over time. In the MCI sample, we expected that individuals with self‐reported hearing loss would also do significantly worse over time on a global assessment of cognition. However, our results revealed no significant difference between participants with and without hearing loss on longitudinal ADAS Cog‐11 performance. This observation may reflect differences in the cognitive domains captured between these composite cognitive measures. One interpretation is that the association between hearing impairment and longitudinal changes in cognition may only be observable within individuals with normal cognition, with other sources (eg, disease pathology) obscuring the impact of hearing loss once an individual becomes symptomatic. However, other studies demonstrated that hearing loss was associated with impairment across multiple cognitive domains in individuals with MCI.^39^, ^40^ Finally, in both our CN and MCI samples, individuals with self‐reported hearing loss scored significantly worse on the FAQ, consistent with findings that show hearing loss is associated with worse independent functioning longitudinally.^41^ This association was independent of cognition, suggesting that the relationship may be attributable to the hearing loss independent of potential cognitive changes arising from hearing impairment.^42^

In addition, exploratory analyses examined the impact of hearing loss on specific cognitive domains (Table S5). Cognitively normal individuals with hearing loss performed significantly worse on the CDR‐sb, MMSE, RAVLT immediate, RAVLT percent forgetting, and ADNI‐Mem composite scores. In the MCI sample with hearing loss, individuals also performed significantly worse on the RAVLT percent forgetting, LMII, and ADNI‐Mem scores. These findings suggest that tests of memory are affected by hearing loss over time. Although exploratory, these results are in line with previous publications demonstrating increased rates of cognitive decline in those with hearing loss.^4^, ^6^, ^13^, ^14^

Interestingly, we did not find self‐reported hearing loss to be associated with a higher risk of converting to a more impaired diagnostic group. These findings are inconsistent with previous literature reporting hearing loss as a risk for future cognitive decline and dementia.^4^, ^6^, ^7^, ^13^, ^16^ However, our results are consistent with other studies,^5^ including large‐scale data sets,^43^, ^44^ which found hearing loss was not significantly related to diagnostic conversion. Taken together, these results suggest that greater risk of conversion to a more impaired diagnosis may not be a direct effect of hearing loss but may instead be mediated by other factors.

When examining these findings, the question as to the relationship between hearing loss and raw testing scores should be considered. An inability to hear verbal instructions and stimuli of the cognitive tests could explain some of the observed group differences, which is especially true for the baseline cognitive testing. However, if the observed differences between those with and without hearing loss were only related to the inability to hear verbal cues, we would not expect to find a significant interaction of hearing loss over time on multiple cognitive and functional outcomes, as such results imply divergent slopes over time. Cognitively normal individuals with hearing loss have also been found to perform similarly on cognitive assessments whether testing was administered through auditory or visual modalities.^45^

### 4.3 Limitations and Future Directions

There are several limitations to this study. While we did not demonstrate that hearing loss increased the risk of conversion to a more impaired diagnostic group, the ADNI dataset currently has relatively few individuals enrolled in the study with longitudinal comprehensive cognitive testing for longer than 96 months (Supplemental Table S1). As such, our analysis may be underpowered to accurately assess the risk of hearing loss on clinical conversion, especially if this risk requires several years to demonstrate a measurable effect.

A noted limitation to these analyses relates to how hearing loss was queried and documented at sites participating in ADNI. This study did not specifically or directly ask whether hearing loss was present, nor were any objective measurements of hearing acuity collected. All medical history was collected through self‐report or caregiver report and as such is subject to recall bias, which may have resulted in an underestimation of the true prevalence of hearing loss. However, as we still demonstrated a significant association between hearing loss and longitudinal cognitive/functional performance, our findings may have been even more robust if the presence/absence of hearing loss could have been more accurately assessed. Further, secondary to inconsistent reporting, we were unable to control for any potential treatment effect conferred by hearing aid use. Future studies should evaluate the relationship between hearing loss and diagnostic conversion over longer periods of time with larger samples. Additionally, future iterations of large‐scale observational protocols (ADNI4) could be modified to collect more detailed information about hearing loss and hearing aid use. Finally, given the known sex‐based differences in the prevalence of hearing loss,^33^, ^34^ future investigations should be made into potential sex‐based differences on the longitudinal association between hearing loss and cognition/function.^46^

Given the increasing evidence that subjective memory concerns may be a risk factor for future cognitive decline,^47^ the inclusion of participants with subjective memory concerns in the CN cohort limited the ability to determine whether participants with subjective memory concerns and comorbid hearing loss are at an even greater risk of cognitive and functional decline. Finally, it would be of great interest for future studies to investigate the association between hearing loss and different etiologies and stages (including preclinical Alzheimer's disease) of cognitive impairment.

A more comprehensive understanding of the potentially modifiable risk imparted by hearing loss on cognitive impairment and daily functioning may also be of current public concern, as a criterion for recently approved disease‐modifying therapies for Alzheimer's disease is a diagnosis of MCI or early dementia. Hearing loss may be a factor in cognitive and functional performance and should be taken into consideration when determining the clinical eligibility to receive these newly approved therapies.

## 5 CONCLUSION

Overall, our findings in the ADNI sample of convenience add evidence to the existing body of literature demonstrating that hearing loss contributes to faster decline across a variety of cognitive and functional outcomes in both cognitively normal participants and those with MCI. In this dataset, we did not demonstrate an increased risk of conversion to a more impaired diagnosis in those participants with hearing loss.

## CONFLICT OF INTEREST STATEMENT

R.S.O. reports grants for clinical trials from Cognition Therapeutics and Bristol‐Myers Squibb outside of the submitted work. A.P.M. reports grants for clinical trials from Genentech, Eli Lilly, and Janssen Pharmaceuticals outside the submitted work. C.H.v.D. reports grants for clinical trials from Biogen, Novartis, Eli Lilly, Merck, Eisai, Janssen, Roche, Genentech, Toyama, and Biohaven outside the submitted work. A.P.M., E.S.S., Y.Z., and C.H.v.D. report grant support from the NIH for work not related to this manuscript. C.H.v.D. reports consulting fees from Kyowa Kirin, Roche, Merck, Eli Lilly, and Janssen. A.P.M. received honoraria for presentations at University of Connecticut and Stanford University. R.S.O. received honoraria for presentations at University of Kansas. A.P.M. received support from ACTC/ATRI for travel to ACTC/ATRI meetings. A.P.M. is a member of the ISTAART Neuroimaging PIA executive committee. A.A.M. and S.W. have no conflicts of interest to disclose. Author disclosures are available in the Supporting Information.

## SOURCES OF FUNDING

This work was supported by the National Institute on Aging (P30AG066508, P30AG021342, RF1AG081413, and RF1AG068191). Its contents are solely the responsibility of the authors and do not necessarily represent the official view of NIA/NIH. The funders had no role in the design and conduct of the study; collection, management, analysis, and interpretation of the data; preparation, review, or approval of the manuscript; and decision to submit the manuscript for publication.

## CONSENT STATEMENT

All participants gave their informed consent, and the study protocol was approved by the committee on human research at each participating institution.

## Supporting information



Supporting Information

Supporting Information
