# Supplementary material for: Self‐reported hearing loss is associated with faster cognitive and functional decline but not diagnostic conversion in the ADNI cohort
Source: Alzheimers Dement. 2024 Sep 26;20(11):7847–58. doi: 10.1002/alz.14252 (PMC11567835; doi:10.1002/alz.14252)
Supplement: Supplementary file 1 — Supporting Information [file ALZ-20-7847-s001.docx]

**Self-Reported Hearing Loss is Associated with Faster Cognitive and Functional Decline but not Diagnostic Conversion in the ADNI Cohort**

Alyssa A. Miller^a,b^, Emily S. Sharp^a,c^, Selena Wang^d,e^, Yize Zhao^d^, Adam P. Mecca^a,b^, Christopher H. van Dyck^a-c,e^, Ryan S. O’Dell^a,b^, The Alzheimer’s Disease Neuroimaging Initiative (ADNI)

^a^Alzheimer’s Disease Research Unit, Yale University School of Medicine, One Church Street, 8^th^ Floor, New Haven, CT, 06510, USA

^b^Department of Psychiatry, Yale University School of Medicine, 300 George Street, Suite 901, New Haven, CT, 06510, USA

^c^Department of Neurology, Yale University School of Medicine, P.O. Box 208018, New Haven, CT, 06520 USA

^d^Department of Biostatistics, Yale University School of Public Health, 60 College Street, New Haven, CT, 06510 USA.

^e^Department of Biostatistics and Health Data Science, Indiana University School of Medicine, 410 W. 10^th^ Street, Indianapolis, IN, 46202 USA.

^f^Department of Neuroscience, Yale University School of Medicine, P.O. Box 208001, New Haven, CT, 06520, USA

For correspondence or reprints contact:

Ryan S. O’Dell, M.D., Ph.D.

Alzheimer’s Disease Research Unit

Yale University School of Medicine

One Church Street, 8^th^ Floor

New Haven, CT 06510

tel +1 203 764-8100

fax +1 203 764-8111

Email: ryan.odell@yale.edu

**Running Title:** Hearing loss and cognitive decline in ADNI

**Table of Contents:**

| **Content** | **Pages** |
| --- | --- |
| Supplemental Table 1. Number of observations (n) for primary outcome measures at each visit by baseline diagnosis and hearing loss status | 3 - 4 |
| Supplemental Table 2. Main Effects of Baseline Demographic Variables on mPACC in CN Participants | 5 |
| Supplemental Table 3. Main Effects of Baseline Demographic Variables on ADAS-Cog-11 in MCI Participants | 6 |
| Supplemental Table 4. Main Effects of Baseline Demographic Variables on FAQ in CN and MCI Participants | 7 |
| Supplemental Table 5. Association Between Hearing Loss and Change in Cognition and Function Over Time – Exploratory Outcomes | 8 |
| Supplemental Figure 1. Change in cognition and function over time for cognitively normal participants with and without hearing loss | 9 |
| Supplemental Figure 2. Change in cognition and function over time for MCI participants with and without hearing loss. | 10 |

| **Supplemental Table 1. Number of observations (n) for primary outcome measures at each visit by baseline diagnosis and hearing loss status** | | | | | | |
| --- | --- | --- | --- | --- | --- | --- |
| **Visit (months)** | **CN at baseline** | | | **MCI at baseline** | | |
|  | **mPACC** | **ADAS-Cog-11** | **FAQ** | **mPACC** | **ADAS-Cog-11** | **FAQ** |
| **0** | 695 (561,134) | 694 (560,134) | 695 (561,134) | 941 (730,211) | 939 (728,211) | 931 (723,208) |
| **6** | 474 (370,104) | 474 (370,104) | 471 (368,103) | 777 (601,176) | 776 (600,176) | 775 (599,176) |
| **12** | 458 (364,94) | 457 (363,94) | 455 (362,93) | 877 (679,198) | 875 (678,197) | 868 (674,194) |
| **24** | 551 (439,112) | 545 (434,111) | 537 (426,111) | 707 (546,161) | 705 (544,161) | 704 (544,160) |
| **36** | 232 (185,47) | 231 (184,47) | 228 (182,46) | 588 (459,129) | 586 (458,128) | 584 (454,130) |
| **48** | 316 (236,80) | 315 (235,80) | 306 (233,73) | 388 (302,86) | 386 (301,85) | 383 (297,86) |
| **60** | 128 (94,34) | 127 (93,34) | 127 (94,33) | 224 (169,55) | 224 (168,56) | 221 (167,54) |
| **72** | 161 (123,38) | 162 (124,38) | 160 (123,37) | 163 (120,43) | 163 (120,43) | 163 (120,43) |
| **84** | 109 (83,26) | 109 (83,26) | 108 (82,26) | 127 (97,30) | 125 (95,30) | 126 (96,30) |
| **96** | 93 (70,23) | 93 (70,23) | 93 (71,22) | 89 (69,20) | 89 (69,20) | 88 (68,20) |
| **108** | 63 (52,11) | 63 (52,11) | 60 (49,11) | 57 (41,16) | 55 (39,16) | 55 (39,16) |
| **120** | 56 (43,13) | 56 (43,13) | 55 (42,13) | 40 (33,7) | 39 (32,7) | 41 (33,8) |
| **132** | 27 (18,9) | 27 (18,9) | 27 (18,9) | 17 (13,4) | 17 (13,4) | 17 (13,4) |
| **144** | 19 (16,3) | 19 (16,3) | 17 (15,2) | 7 (5,2) | 7 (5,2) | 9 (6,3) |
| **150** | na | na | na | 6 (5,1) | 6 (5,1) | 6 (5,1) |
| **156** | 21 (15,6) | 21 (15,6) | 20 (14,6) | na | na | na |
| **168** | 4 (3,1) | 4 (3,1) | 4 (3,1) | na | na | na |
| **174** | na | na | na | 2 (1,1) | 2 (1,1) | 2 (1,1) |
| **180** | 10 (8,2) | 10 (8,2) | 10 (8,2) | na | na | na |
| **186** | na | na | na | 2 (1,1) | 1 (0,1) | 2 (1,1) |

Data are the total n for the primary outcome measures at each visit in participants with diagnoses of CN and MCI at baseline. Enclosed in parentheses are n for participants with no hearing loss and n for participants with hearing loss, respectively. Visit number is denoted as months from baseline. Visit 0 represents baseline. Abbreviations: CN, cognitive normal; MCI, mild cognitive impairment; mPACC, modified Preclinical Alzheimer’s Cognitive Composite; ADAS-Cog-11, Alzheimer’s Disease Assessment Scale-Cognitive Sub-Score; FAQ, Functional Activities Questionnaire.

| **Supplemental Table 2. Main Effects of Baseline Demographic Variables on mPACC in CN Participants** | | | |
| --- | --- | --- | --- |
|  | **F** | **Parameter Estimate** | ***P*** |
| **Hearing Loss (present)** | 3.42 | 0.48 | 0.065 |
| **Time (months)** | 327.16 | -0.025 | < 0.001* |
| **Age** | 55.99 | -0.12 | < 0.001* |
| **Sex (female)** | 2.70 | 0.34 | 0.10 |
| **Education (years)** | 41.49 | 0.24 | < 0.001* |
| **APOE ɛ4 – 0 copies** | 2.88 | 0.62 | 0.30 |
| **APOE ɛ4 – 1 copy** | 2.88 | 0.14 | 0.82 |
| **Baseline ADAS-Cog-11** | 159.05 | -0.44 | < 0.001* |

To determine if differences in cognitive decline were associated with the presence of hearing loss, separate repeated-measures liner mixed models were used with mPACC score as the outcome variable and the interaction of hearing loss and time as the main explanatory variable. Age, sex, education, APOE ɛ4 copy number, baseline ADAS-Cog-11, and a random intercept were included as covariates. For participants with a diagnosis of CN at baseline, the primary cognitive outcome was the mPACC. F statistics, parameter estimates, and *P* values are reported here for the main effects of hearing loss, time (in months), age (in years), female sex, education, APOE ɛ4 copy number, and baseline ADAS-Cog-11 on mPACC in CN participants. The results of the main explanatory variable for this model (the interaction term of hearing loss*time on mPACC in CN participants) are reported in **Table 2**. Time is documented in months for this model. * *P* < 0.05. Abbreviations: CN, cognitive normal; mPACC, modified Preclinical Alzheimer’s Cognitive Composite; ADAS-Cog-11, Alzheimer’s Disease Assessment Scale-Cognitive Sub-Scale; APOE, apolipoprotein.

| **Supplemental Table 3. Main Effects of Baseline Demographic Variables on ADAS-Cog-11 in MCI Participants** | | | |
| --- | --- | --- | --- |
|  | **F** | **Parameter Estimate** | ***P*** |
| **Hearing Loss (present)** | 0.16 | 0.14 | 0.69 |
| **Time (months)** | 609.02 | 0.07 | < 0.001* |
| **Age** | 18.48 | 0.08 | < 0.001* |
| **Sex (female)** | 12.83 | 0.96 | < 0.001* |
| **Education (years)** | 0.17 | -0.02 | 0.68 |
| **APOE ɛ4 – 0 copies** | 13.37 | -2.40 | < 0.001* |
| **APOE ɛ4 – 1 copy** | 13.37 | -1.07 | 0.016* |
| **Baseline ADAS-Cog-11** | 1318.18 | 1.08 | < 0.001* |

To determine if differences in cognitive decline were associated with the presence of hearing loss, separate repeated-measures liner mixed models were used with ADAS-Cog-11 score as the outcome variable and the interaction of hearing loss and time as the main explanatory variable. Age, sex, education, APOE ɛ4 copy number, baseline ADAS-Cog-11, and a random intercept were included as covariates. For participants with a diagnosis of MCI at baseline, the primary cognitive outcome was the ADAS-Cog-11. F statistics, parameter estimates, and *P* values are reported here for the main effects of hearing loss, time (in months), age (in years), female sex, education, APOE ɛ4 copy number, and baseline ADAS-Cog-11 on ADAS-Cog-11 in MCI participants. The results of the main explanatory variable for this model (the interaction term of hearing loss*time on ADAS-Cog-11 in MCI participants) are reported in **Table 2**. Time is documented in months for this model. * *P* < 0.05. Abbreviations: MCI, mild cognitive impairment; ADAS-Cog-11, Alzheimer’s Disease Assessment Scale-Cognitive Sub-Scale; APOE, apolipoprotein.

| **Supplemental Table 4. Main Effects of Baseline Demographic Variables on FAQ in CN and MCI Participants** | | | | | |  |  |
| --- | --- | --- | --- | --- | --- | --- | --- |
|  | **CN at baseline** | | | **MCI at baseline** | | | |
|  | **F** | **Parameter Estimate** | ***P*** | **F** | **Parameter Estimate** | | ***P*** |
| **Hearing Loss (present)** | 0.013 | 0.024 | 0.91 | 0.092 | 0.053 | | 0.76 |
| **Time (months)** | 331.56 | 0.022 | < 0.0001* | 248.83 | 0.023 | | < 0.0001* |
| **Age** | 8.82 | 0.039 | 0.003* | 30.52 | 0.051 | | < 0.0001* |
| **Sex (female)** | 0.17 | 0.067 | 0.68 | 2.75 | 0.180 | | 0.10 |
| **Education (years)** | 1.25 | -0.033 | 0.26 | 6.37 | -0.050 | | 0.012* |
| **APOE ɛ4 – 0 copies** | 0.12 | 0.009 | 0.99 | 1.18 | 0.025 | | 0.94 |
| **APOE ɛ4 – 1 copy** | 0.12 | 0.092 | 0.85 | 1.18 | 0.201 | | 0.57 |
| **Baseline ADAS-Cog-11** | 4.75 | 0.059 | 0.03* | 15.26 | 0.071 | | < 0.0001* |

To determine if differences in functional decline were associated with the presence of hearing loss, separate repeated-measures liner mixed models were used with FAQ score as the outcome variable and the interaction of hearing loss and time as the main explanatory variable. Age, sex, education, APOE ɛ4 copy number, baseline ADAS-Cog-11, and a random intercept were included as covariates. For participants with either a diagnosis of CN or MCI at baseline, the primary functional outcome was the FAQ. F statistics, parameter estimates, and *P* values are reported here for the main effects of hearing loss, time (in months), age (in years), female sex, education, APOE ɛ4 copy number, and baseline ADAS-Cog-11 on FAQ in CN and MCI participants. The results of the main explanatory variable for these models (the interaction term of hearing loss*time on FAQ in CN and MCI participants) are reported in **Table 2**. Time is documented in months for this model. * *P* < 0.05. Abbreviations: CN, cognitive normal; MCI, mild cognitive impairment; mPACC, modified Preclinical Alzheimer’s Cognitive Composite; ADAS-Cog-11, Alzheimer’s Disease Assessment Scale-Cognitive Sub-Scale; APOE, apolipoprotein.

| **Supplemental Table 5. Association Between Hearing Loss and Change in Cognition and Function Over Time – Exploratory Outcomes** | | | | | | |
| --- | --- | --- | --- | --- | --- | --- |
|  | **CN at Baseline** | | | **MCI at Baseline** | | |
|  | **F** | **Parameter Estimate** | ***P*** | **F** | **Parameter Estimate** | ***P*** |
| **CDRsb** | 7.30 | 0.003 | 0.007* | 0.68 | 0.002 | 0.41 |
| **MMSE** | 13.74 | -0.007 | <0.001* | 1.12 | -0.003 | 0.29 |
| **RAVLT Immediate** | 6.18 | -0.020 | 0.013* | 1.73 | -0.009 | 0.19 |
| **RAVLT Learning** | 0.01 | -0.0002 | 0.94 | 0.002 | -0.0001 | 0.96 |
| **RAVLT Percent Forgetting** | 5.60 | 0.076 | 0.018* | 7.61 | 0.126 | 0.006* |
| **Logical Memory Delayed Recall** | 1.59 | -0.005 | 0.21 | 39.32 | -0.022 | <0.001* |
| **Trails B** | 0.00 | -0.00005 | 0.99 | 0.80 | 0.054 | 0.37 |
| **Digit Symbol Substitution Test** | 1.85 | 0.038 | 0.17 | 3.67 | 0.044 | 0.056 |
| **ADNI-Mem** | 4.19 | -0.001 | 0.041* | 4.29 | -0.001 | 0.038* |
| **ADNI-EF** | 0.004 | -0.00004 | 0.95 | 1.96 | -0.0009 | 0.16 |

To determine if differences in cognitive and functional decline were associated with the presence of hearing loss, separate repeated-measures liner mixed models were used with different cognitive and functional measures as the outcome variable and the interaction of hearing loss and time as the main explanatory variable. Age, sex, education, APOE ɛ4 copy number, baseline ADAS-Cog-11, and a random intercept were included as covariates. Separate models were used for participants with baseline diagnoses of CN and MCI. F statistics, parameter estimates, and *P* values are reported for the main explanatory variable of the hearing loss*time interaction term. Time is documented in months for these models. * *P* < 0.05. Abbreviations: CN, cognitive normal; MCI, mild cognitive impairment; CDR-sb, Clinical Dementia Rating scale – sum of boxes; MMSE, Mini Mental Status Exam; ADAS-Cog-11, Alzheimer’s Disease Assessment Scale-Cognitive Sub-Score; RAVLT- Rey auditory verbal learning test; ADNI-Mem, composite memory score; ADNI-EF, composite executive function score.

**Supplemental Figure 1. Change in cognition and function over time for cognitively normal participants with and without hearing loss.** Change in adjusted CDRsb (A), MMSE (B), RAVLT immediate (C), RAVLT learning (D), RAVLT percent forgetting (E), Logical Memory delayed recall (F), Trails B (G), Digit Symbol Substitution Test (H), ADNI Mem (I), and ADNI EF (J) scores with 95% CI over time in participants with a baseline diagnosis of CN. Repeated-measures linear mixed models with cognitive performance (A-J) as the outcome variable and the interaction of hearing loss and time as the main explanatory variable were used to determine the relationship between hearing loss,cognition, and time. Age, sex, education, APOE ɛ4 copy number, baseline ADAS-Cog-11, and a random intercept were included as covariates. Adjusted scores represent an estimated mean when sex = male, APOE ɛ4 copy number = 2, baseline ADAS-Cog-11 = 0, age = 0, and education = 0. Parameter estimates (PE) and *P* values are reported for the main explanatory variable of the hearing loss*time interaction term from the original linear mixed models. Abbreviations: CN, cognitive normal; ADAS-Cog-11, Alzheimer’s Disease Assessment Scale-Cognitive Sub-Score; CDR-sb, Clinical Dementia Rating scale – sum of boxes; MMSE, Mini Mental Status Exam; RAVLT- Rey auditory verbal learning test; ADNI-Mem, composite memory score; ADNI-EF, composite executive function score; PE, parameter estimate; CI, confidence interval.

**Supplemental Figure 2. Change in cognition and function over time for MCI participants with and without hearing loss.** Change in adjusted CDRsb (A), MMSE (B), RAVLT immediate (C), RAVLT learning (D), RAVLT percent forgetting (E), Logical Memory delayed recall (F)­­­, Trails B (G), Digit Symbol Substitution Test (H), ADNI Mem (I), and ADNI EF (J) scores with 95% CI over time in participants with a baseline diagnosis of MCI. Repeated-measures linear mixed models with cognitive performance (A-J) as the outcome variable and the interaction of hearing loss and time as the main explanatory variable were used to determine the relationship between hearing loss, cognition, and time. Age, sex, education, APOE ɛ4 copy number, baseline ADAS-Cog-11, and a random intercept were included as covariates. Adjusted scores represent an estimated mean when sex = male, APOE ɛ4 copy number = 2, baseline ADAS-Cog-11 = 0, age = 0, and education = 0. Parameter estimates (PE) and *P* values are reported for the main explanatory variable of the hearing loss*time interaction term from the original linear mixed models. Abbreviations: MCI, mild cognitive impairment; ADAS-Cog-11, Alzheimer’s Disease Assessment Scale-Cognitive Sub-Score; CDR-sb, Clinical Dementia Rating scale – sum of boxes; MMSE, Mini Mental Status Exam; RAVLT- Rey auditory verbal learning test; ADNI-Mem, composite memory score; ADNI-EF, composite executive function score; PE, parameter estimate; CI, confidence interval.
